# A hybrid model for hand-foot-mouth disease prediction based on ARIMA-EEMD-LSTM

**DOI:** 10.1186/s12879-023-08864-y

**Published:** 2023-12-15

**Authors:** Yiran Wan, Ping Song, Jiangchen Liu, Ximing Xu, Xun Lei

**Affiliations:** 1 School of Public Health, Chongqing Medical University, Chongqing, China; 2 Research Center for Medicine and Social Development, Chongqing, China; 3 Collaborative Innovation Center of Social Risks Governance in Health, Chongqing Medical University, Chongqing, China; 4 Research Center for Public Health Security, Chongqing Medical University, No1 Medical College Rd, Yuzhong District, Chongqing, 400016 People’s Republic of China; 5 Big Data Center for Children’s Medical Care, Children’s Hospital of Chongqing Medical University, National Clinical Research Center for Child Health and Disorders, Ministry of Education Key Laboratory of Child Development and Disorders, No 136. Zhongshan 2Nd Rd, Yuzhong District, Chongqing, 400014 People’s Republic of China; 6 School of Mathematical Science, Chongqing Normal University, Chongqing, China

**Keywords:** EEMD, LSTM, Hybrid model, HFMD prediction

## Abstract

**Background:**

Hand, foot, and mouth disease (HFMD) is a common infectious disease that poses a serious threat to children all over the world. However, the current prediction models for HFMD still require improvement in accuracy. In this study, we proposed a hybrid model based on autoregressive integrated moving average (ARIMA), ensemble empirical mode decomposition (EEMD) and long short-term memory (LSTM) to predict the trend of HFMD.

**Methods:**

The data used in this study was sourced from the National Clinical Research Center for Child Health and Disorders, Chongqing, China. The daily reported incidence of HFMD from 1 January 2015 to 27 July 2023 was collected to develop an ARIMA-EEMD-LSTM hybrid model. ARIMA, LSTM, ARIMA-LSTM and EEMD-LSTM models were developed to compare with the proposed hybrid model. Root mean square error (RMSE), mean absolute error (MAE) and coefficient of determination (R^2^) were adopted to evaluate the performances of the prediction models.

**Results:**

Overall, ARIMA-EEMD-LSTM model achieved the most accurate prediction for HFMD, with RMSE, MAPE and R^2^ of 4.37, 2.94 and 0.996, respectively. Performing EEMD on the residual sequence yields 11 intrinsic mode functions. EEMD-LSTM model is the second best, with RMSE, MAPE and R^2^ of 6.20, 3.98 and 0.996.

**Conclusion:**

Results showed the advantage of ARIMA-EEMD-LSTM model over the ARIMA model, the LSTM model, the ARIMA-LSTM model and the EEMD-LSTM model. For the prevention and control of epidemics, the proposed hybrid model may provide a more powerful help. Compared with other three models, the two integrated with EEMD method showed significant improvement in predictive capability, offering novel insights for modeling of disease time series.

**Supplementary Information:**

The online version contains supplementary material available at 10.1186/s12879-023-08864-y.

## Background

Hand, foot, and mouth disease (HFMD) is a common infectious disease caused by a group of enteroviruses, particularly among children under the age of 5 [1, 2]. The main symptoms of HFMD are fever, rashes and ulcers in the hand, foot, or oral mucosa [3]. Although HFMD tends to be mild and self-limiting in the majority of patients, it can lead to neurological complications, pulmonary edema, and even death in severe cases [4].

A high prevalence of HFMD has been reported in Asia [5]. During the past two decades, HFMD outbreaks were reported frequently from Asian countries such as Japan [6], Malaysia [7], Singapore [8] and Vietnam [9]. In China, the number of HFMD confirmed cases has been the first among all the statutory reported infectious diseases since it was included in the management of Category C statutory infectious diseases in 2008 [10].

Accurate prediction and early warning for the trend of HFMD provides valuable insights for the rational allocation of medical resources and assist in the prevention and control of HFMD. Since the outbreak of HFMD, researchers around the world have conducted numerous studies and developed various prediction models. Autoregressive integrated moving average (ARIMA) model, which has been widely used for epidemic prediction during the past decades, has been built based on the historical data from China [11, 12] and Malaysia [7]. However, as a linear model, ARIMA is not good at extracting non-linear features. Deep learning algorithms perform better when deal with non-linear features of time series. Long short-term memory (LSTM) model is one of the most popular deep learning algorithms, which has been increasingly used for epidemic prediction in recent years [11, 13]. To further improve the prediction accuracy, a variety of hybrid models have been proposed. The combination of ARIMA and LSTM combines the advantages of both linear and non-linear models to achieve higher accuracy, which has been applied in various fields such as environmental science [14] and epidemic prediction [15].

Empirical Mode Decomposition (EMD) is a data analysis method that has gained attention in various fields for its ability to decompose complex and nonstationary signals [16]. However, EMD suffers from mode mixing and end effects, which limit its applicability. In order to address these issues, Ensemble Empirical Mode Decomposition (EEMD) was proposed as an improved version of EMD. EEMD overcomes the limitations of EMD by adding a noise-assisted step to the decomposition process [17]. It involves repeatedly adding white noise to the original signal and decomposing the resulting ensemble of signals using EMD. By averaging the obtained intrinsic mode functions (IMFs) across the ensemble, EEMD effectively reduces mode mixing and suppresses the end effects. It provides a valuable tool for analyzing and extracting meaningful information from complex and nonlinear data. By decomposing the data into simpler and more suitable components, EEMD allows for better understanding of the underlying patterns and facilitates the application of subsequent analysis techniques.

In this study, we trained a hybrid ARIMA-EEMD-LSTM model based on the daily incidence of HFMD extracted from the largest pediatric hospital in Chongqing, China from 2015 to 2021, to predict the incidence of HFMD in 2022 and 2023. The proposed hybrid model is expected to provide more accurate predictions and better decision-making support for disease prevention and control.

## Materials and methods

### Study design and data collection

The data used in this study was obtained from the Clinical Research Data Platform of the Children's Hospital of Chongqing Medical University (the National Clinical Research Center for Child Health and Disorders in China), which contained the clinical information of more than 8,000,000 pediatric outpatients and inpatients as of July 2023. We collected the daily confirmed incidence of HFMD from 1 January 2015 to 27 July 2023. 80% of the data was used as the training set to train the prediction models, and the remaining 20% was used as the test set to evaluate model performance.

### Autoregressive integrated moving average (ARIMA)

ARIMA is one of the classical methods of time series prediction analysis, whose main idea is to transform the non-stationary time series into stationary time series by differencing before model development. The process of constructing an ARIMA model is as follows: ADF test is performed on the differenced series to judge its stationarity, determine the number of differences d of the stationary series obtained, and determine the parameters p and q by autocorrelation function (ACF) and partial autocorrelation function (PACF), and then get the ARIMA (p, d, q) model.

### Ensemble empirical mode decomposition (EEMD)

Empirical mode decomposition (EMD) is a method proposed by E. Huang for the analysis and processing of non-linear and non-stationary signals, which considers that any signal can be split into several intrinsic mode functions (IMF) and a residual component [16]. The EMD decomposition is given as

$${\mathrm{X}}_{(\mathrm{t})}={\sum }_{\mathrm{i}=1}^{\mathrm{n}}{\mathrm{c}}_{\mathrm{i}}(\mathrm{t})+\mathrm{r}(\mathrm{t})$$

${\mathrm{X}}_{(\mathrm{t})}$ is the original time series, ${\mathrm{c}}_{\mathrm{i}}(\mathrm{t})$ is the i^th^ IMF, r(t) is the residual.

However, the EMD method has some limitations because the IMFs obtained from its decomposition suffer from mode mixing. In order to solve this problem, Huang E and his team developed an improved EMD method based on noise-assisted analysis, named ensemble empirical mode decomposition (EEMD) [17].

The essence of the EEMD method is a multiple EMD with superimposed Gaussian white noise, which takes advantage of the statistical property of Gaussian white noise with uniform frequency distribution to change the polar characteristics of the signal by adding different white noise of the same amplitude each time, and then the corresponding IMF obtained from the multiple EMD is averaged to cancel the added white noise, thus effectively suppressing the generation of modal aliasing.

### Long short-term memory (LSTM)

**Fig. 1:**
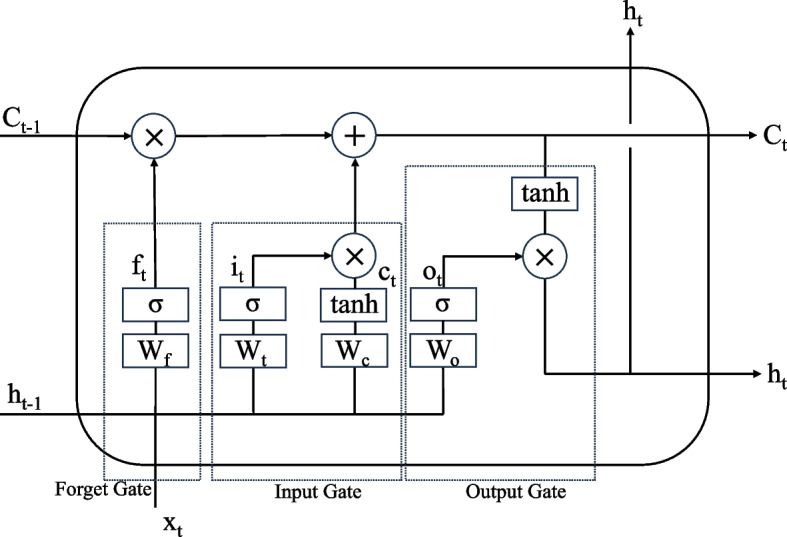
Architecture of long short-term memory

Long Short-Term Memory, is a special type of recurrent neural network (RNN) model that effectively addresses the issue of long-term dependencies commonly encountered in conventional RNN models. LSTM enhances the performance of the traditional RNN architecture by incorporating a more complex cell structure in the hidden layer. In contrast to the regular RNN, LSTM introduces three gate controllers, namely the input gate, forget gate, and output gate, which are controlled by sigmoid functions and combined with the tanh function. Additionally, a summation operation is applied to reduce the possibility of gradient vanishing or exploding. These modifications enable the LSTM neural network to maintain longer-term memory capacity, as illustrated in Fig. 1.

The previous short-term memory h_t-1_ and the current input features x_t_ undergo the forget gate f_t_, input gate (update gate) i_t_, and $\widetilde{c}$ (a candidate for the new cell state), followed by the output gate o_t_, and then normalized through activation functions. The formulas are as follows:

$$\begin{array}{c}{f}_{t}=\sigma ({W}_{f}\left[{h}_{t-1},{x}_{t}\right]+{b}_{f})\\ {i}_{t}=\sigma ({W}_{i}\left[{h}_{t-1},{x}_{t}\right]+{b}_{i})\\ \begin{array}{c}{\widetilde{C}}_{t}=tanh({W}_{C}\left[{h}_{t-1},{x}_{t}\right]+{b}_{C})\\ {C}_{t}={f}_{t}*{C}_{t-1}+{i}_{t}*{\widetilde{C}}_{t}\end{array}\end{array}$$

C_t_ is the updated cell state.

$$\begin{array}{c}{o}_{t}=\sigma ({W}_{o}\left[{h}_{t-1},{x}_{t}\right]+{b}_{o})\\ {h}_{t}={o}_{t}*{\mathrm{tanh}(C}_{t})\end{array}$$

h_t_ is the output vector at the current timestep.

### ARIMA-EEMD-LSTM

The distribution of HFMD incidence data along the time dimension exhibits both linear and nonlinear features. The ARIMA model, belonging to linear models, can only capture the linear characteristics in time series data, while the LSTM neural network can compensate for this limitation. Moreover, for complex nonlinear and non-stationary residual sequences, there may exist multiple fluctuation patterns simultaneously, which makes it challenging for the LSTM model to learn their features comprehensively and accurately. The Empirical Mode Decomposition Ensemble (EEMD) method can analyze nonlinear and non-stationary time series by decomposing them into several relatively simpler component sequences, facilitating the LSTM model to learn their patterns effectively.

In this study, we combined the advantages of the ARIMA and LSTM models. The ARIMA model was utilized to capture the linear temporal features in the time series data, while the LSTM model was employed to fit the nonlinear temporal features in the residual sequence. Additionally, the EEMD method was applied to decompose the residual sequence before constructing the LSTM model, thereby improving the prediction performance. The proposed ARIMA-EEMD-LSTM combination model consisted of the following steps: firstly, an ARIMA model was built for the original time series, and the residuals were obtained by subtracting the model's predicted values from the actual values; secondly, the residual sequence was decomposed using the EEMD method into several Intrinsic Mode Function (IMF) components and a trend component; thirdly, individual LSTM models were constructed and used to predict each component sequence, and the predicted values from each model were summed to reconstruct the residual predictions; finally, the ARIMA model's predicted values were adjusted by subtracting the residual predictions, yielding the final predictions of the combination model.

**Fig. 2:**
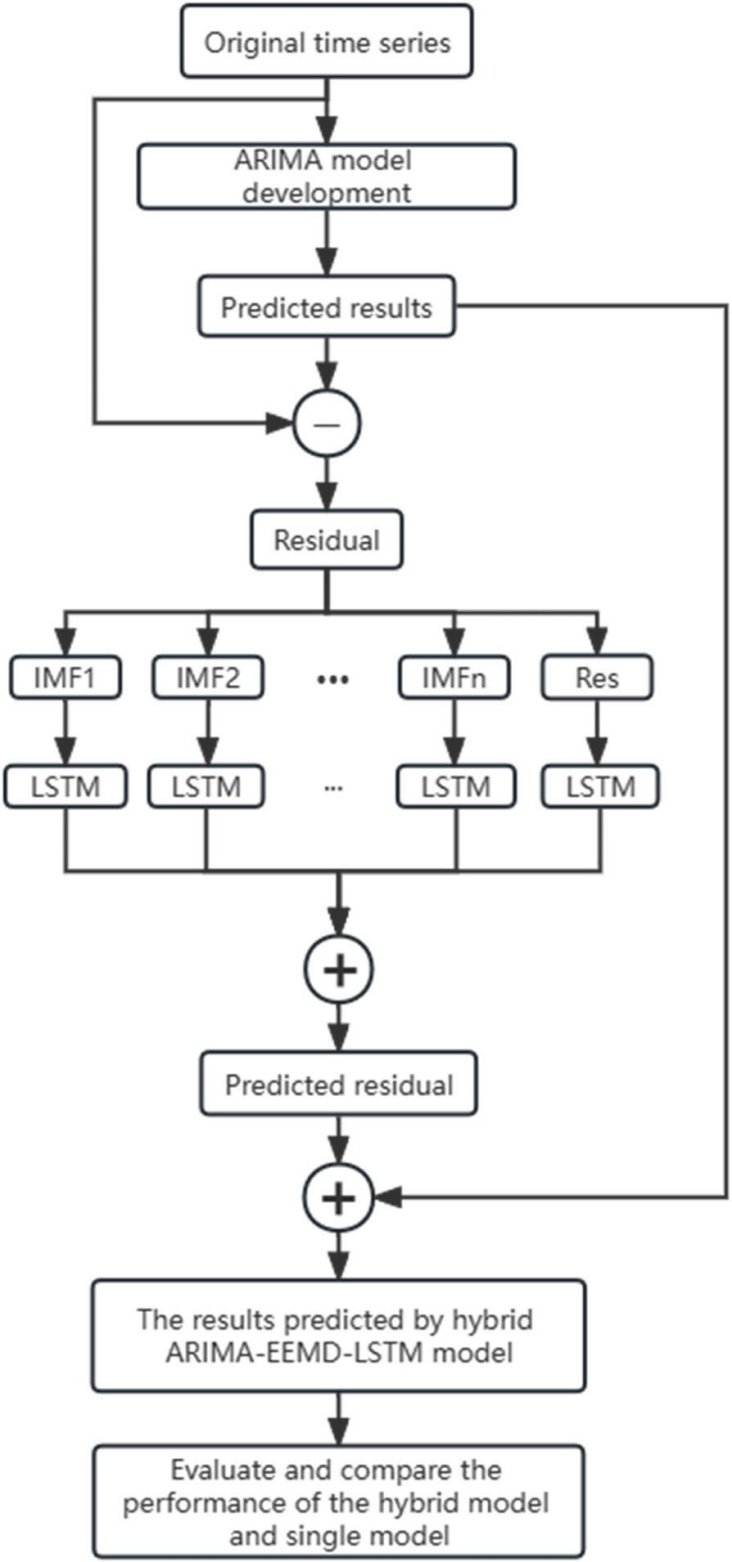
Flowchart of the proposed ARIMA-EEMD-LSTM model

The structure of the ARIMA-EEMD-LSTM combination model is illustrated in Fig. 2.

### Evaluation of model performance

To provide a comprehensive evaluation of the model performance, this study adopted root mean square error (RMSE), mean absolute error (MAE), and coefficient of determination (R^2^), to assess the model performance from different perspectives. The formulas for calculating these metrics are as follows:

$$\mathrm{RMSE}=\sqrt{\frac{1}{\mathrm{n}}\sum_{\mathrm{i}=1}^{\mathrm{n}}{({\widehat{\mathrm{y}}}_{\mathrm{i}}-{\mathrm{y}}_{\mathrm{i}})}^{2}}$$

$$\mathrm{MAE}=\frac{1}{\mathrm{n}}\sum_{\mathrm{i}=1}^{\mathrm{n}}\left|{\widehat{\mathrm{y}}}_{\mathrm{i}}-{\mathrm{y}}_{\mathrm{i}}\right|$$

$${R}^{2}=1-\frac{\sum_{i=1}^{n}{({\widehat{y}}_{i}-{y}_{i})}^{2}}{\sum_{i=1}^{n}{({\overline{y} }_{i}-{y}_{i})}^{2}}$$

${y}_{i}$ is the actual value at the i-th time point, ${\widehat{y}}_{i}$ is the predicted value at the i-th time point, and ${\overline{y} }_{i}$ is the mean value of the actual values.

### Statistical analysis

In this study, we used R for data preprocessing and the developing of ARIMA model, while we employed Python for EEMD decomposition and LSTM model development. For the LSTM model, we set the number of neurons to 100 and the dropout rate to 0.2. We used Mean Squared Error (MSE) as the loss function. Regarding parameters such as batch_size, epoch, and optimizer, we employed a grid search tuning approach to select the best parameter set..

## Results

### The development of ARIMA-EEMD-LSTM

In this study, the original time series was divided into a training set, covering the period from 1 January 2015, to 7 January 2022 (80% of the data), and a testing set, covering the period from 8 January 2022, to 27 July 2023 (20% of the data). A rolling forecast approach was employed, where 60 days of historical data were used to predict the next 1 day.

To begin, the 'forecast' package in R was utilized. The 'auto.arima' function was employed to identify the optimal model parameters for the training data, resulting in the creation of an ARIMA(5,1,2) model. The ARIMA model was fitted to the training set and used to make predictions on the testing set.

**Fig. 3:**
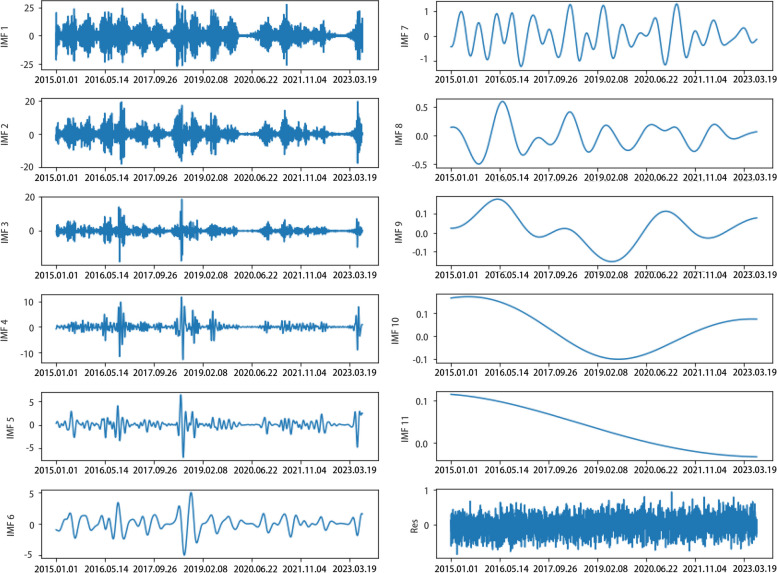
The results of the original data decomposed by EEMD

The EEMD method was applied to decompose the residual series of the ARIMA model, and the results are shown in Fig. 3. The original residual series was decomposed into 11 IMF series and 1 trend series. The IMF series with lower indices represent high-frequency signals in the original sequence, while the IMF series with higher indices represent low-frequency signals. From the decomposition results, it can be observed that the original data contains significant high-frequency signals. When these signals are included in the original time series, they are not easily learned by the LSTM model. However, separating these signals facilitates the learning process for LSTM.

**Fig. 4:**
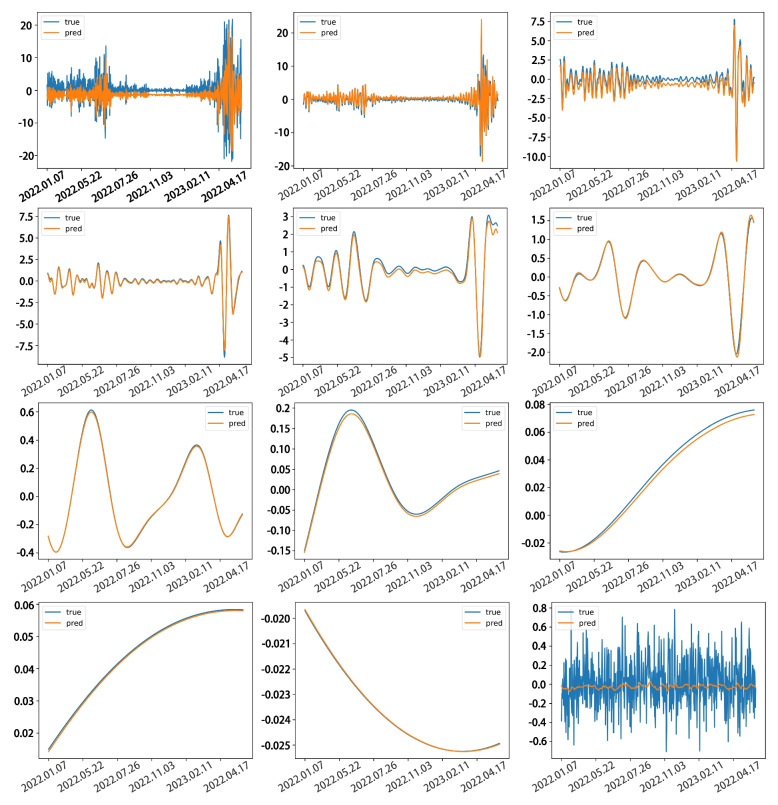
Comparison of predicted values and real values for each IMFs

These decomposed series were used as inputs to train the LSTM models, and the performances of these models on the testing set is shown in Fig. 4. It can be observed that the predicted values of each component series closely match the true values in terms of numerical values and trend, without significant lag.

**Fig. 5:**
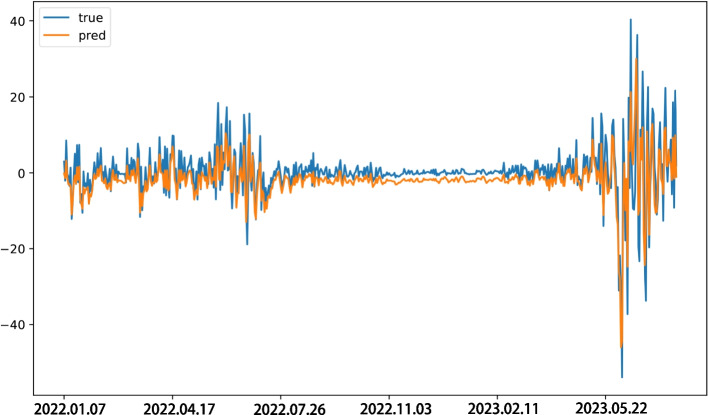
Comparison of predicted values and real values for residual series

The predicted values of the IMF series and the trend series were summed up to obtain the predicted results of the residual series, as shown in Fig. 5. Compared to the actual residual series, the predicted series demonstrates strong consistency in terms of frequency and amplitude of fluctuations, indicating a good predictive effect for the residual series.

**Fig. 6:**
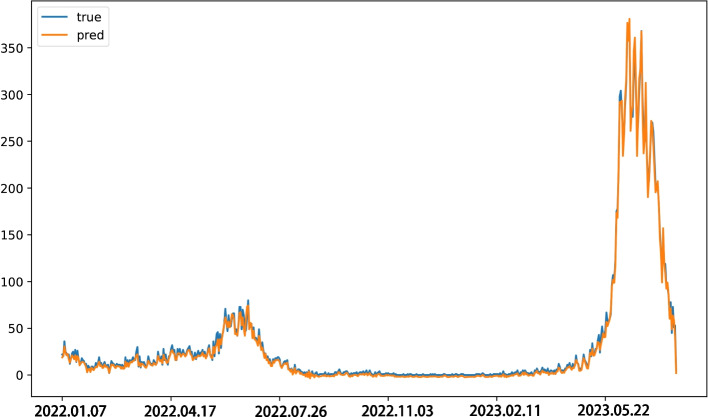
Comparison of predicted values and real values for HFMD confirmed cases

Finally, the predicted values of the ARIMA model and the residual series were added up to obtain the final predicted values, which were compared to the true values in Fig. 6. From the figure, it can be observed that the model accurately predicts the changing trend of the original time series and can capture significant fluctuations.

### The development of other models

In this study, we developed 4 more models as comparison: the ARIMA model, the LSTM model, the ARIMA-LSTM model and the EEMD-LSTM model. The results of those models are shown in Supplemental Figures 1–4.

### Model evaluation and comparison

The evaluation results of the hybrid ARIMA-EEMD-LSTM model, as well as the ARIMA, LSTM, ARIMA-LSTM, and EEMD-LSTM models on the training set and the testing set, are shown in Table 1.

**Table 1:** Comparison of the prediction performances between ARIMA-EEMD-LSTM and other models

Model	Training set			Testing set		
	RMSE	MAE	R^2^	RMSE	MAE	R^2^
ARIMA	9.87	6.78	0.937	6.95	3.68	0.990
LSTM	16.87	12.70	0.820	13.93	8.07	0.961
ARIMA-LSTM	14.42	12.06	0.866	9.85	8.11	0.980
EEMD-LSTM	18.49	14.93	0.780	6.20	3.98	0.992
ARIMA-EEMD-LSTM	6.93	5.35	0.969	4.37	2.94	0.996

The proposed ARIMA-EEMD-LSTM model achieved an RMSE of 4.37, MAE of 2.94, and an R^2^ of 0.996 on the testing set, demonstrating accurate predictions of the incidence of HFMD. In comparison, the ARIMA model had an RMSE of 6.95, MAE of 3.68, and an R^2^ of 0.990, while the LSTM model had an RMSE of 13.93, MAE of 8.07, and an R^2^ of 0.961. The hybrid model outperformed these single models in accuracy and goodness of fit, achieving better predictive performance.

Furthermore, two other hybrid models, ARIMA-LSTM and EEMD-LSTM, were also developed. On the testing set, the ARIMA-LSTM model had an RMSE of 9.85, MAE of 8.11, and an R^2^ of 0.980, while the EEMD-LSTM model had an RMSE of 6.20, MAE of 3.98, and an R^2^ of 0.992. Compared with the LSTM model, EEMD-LSTM showed improvements in RMSE from 13.93 to 6.20, MAE from 8.07 to 3.98, and R^2^ from 0.961 to 0.992. Compared with the ARIMA-LSTM model, ARIMA-EEMD-LSTM showed improvements in RMSE from 9.85 to 4.37, MAE from 8.11 to 2.94, and R^2^ from 0.980 to 0.996. These results indicate that the inclusion of the EEMD method significantly enhances the predictive performance of the models.

Overall, the hybrid ARIMA-EEMD-LSTM model demonstrates superior predictive accuracy and fitness compared with the ARIMA, LSTM, ARIMA-LSTM, and EEMD-LSTM models. The addition of the EEMD method contributes to the improvement of the model's predictive performance.

## Discussion

In this study, we proposed a novel hybrid prediction model which combined the strength of linear statistical model, advanced deep learning model and the cutting-edge EEMD technology to achieve accurate prediction for HFMD incidence. The proposed hybrid ARIMA-EEMD-LSTM model outperformed the other four prediction models developed in this study-ARIMA, LSTM, ARIMA-LSTM and EEMD-LSTM according to the evaluation results, which means the ARIMA-EEMD-LSTM model provides more accurate predictions.

ARIMA, as a classical time series prediction model, has been applied widely in disease predictions [18–20]. However, since belongs to lineal models, ARIMA can only capture the linear characteristics. Many time series in real world contain a mixture of linear and non-linear features, which poses challenges for the predictions of ARIMA model. But the deep learning algorithm can compensate for this limitation. The combination of ARIMA model and LSTM model,the widely used deep learning model for time series,keeps ARIMA’s advantage in capturing linear trends and dependencies within time series while excels at capturing complex,nonlinear patterns and long-term dependencies.

EEMD is a novel technology for processing non-linear and non-stationary data, and has been successfully applied in various fields [21–23]. However, there have been few studies which use EEMD for epidemic predictions. With EEMD method, complex data can be decomposed into relatively simple components that are more suitable for model training. This compensates for the limitation of the LSTM model in dealing with nonstationary time series.

In this study, we compared the hybrid ARIMA-EEMD-LSTM model with two single models-ARIMA and LSTM, and two hybrid models-ARIMA-LSTM and EEMD-LSTM. The evaluation results showed that the ARIMA-EEMD-LSTM model exhibited the best predictive performance with the RMSE, MAPE and R^2^ of 4.37, 2.94 and 0.996, respectively. The predcition performance of the proposed model suggests its potential utility in epidemic prevention and control. And the two models integrated with EEMD method showed significant improvement in predictive capability when compared with other three models. The inclusion of EEMD can have great impact on model performance, offering novel insights for modeling of disease time series.

There are also several limitations in this study. Firstly, the data used in this study were from the National Children's Regional Medical Center (Southwest Region), and more cross-center studies are needed to verify the validity and generalizability of the results. Secondly, models developed in this study only utilized daily cases of HFMD, and more related factors such as temperature and humidity should be considered to furtherly enhance the prediction performance.

## Conclusion

In conclusion, this study proposed an innovative hybrid ARIMA-EEMD-LSTM model for predicting the incidence of HFMD. By integrating the strengths of the ARIMA model, LSTM model, and EEMD method, the hybrid model achieved enhanced prediction accuracy and fit, and can serve as a valuable tool for healthcare professionals and policymakers in understanding and managing the spread of HFMD and other epidemics.

### Supplementary Information

**Additional file 1. **

## Data Availability

The dataset used in the study are available from the corresponding author on reasonable request.

## Abbreviations

HFMD: Hand, foot and mouth disease
ARIMA: Autoregressive integrated moving average
EMD: Empirical mode decomposition
EEMD: Ensemble empirical mode decomposition
LSTM: Long short-term memory
