# Supplementary material for: A hybrid model for hand-foot-mouth disease prediction based on ARIMA-EEMD-LSTM
Source: BMC Infect Dis. 2023 Dec 15;23:879. doi: 10.1186/s12879-023-08864-y (PMC10722819; doi:10.1186/s12879-023-08864-y)
Supplement: Supplementary file 1 — Additional file 1. [file 12879_2023_8864_MOESM1_ESM.docx]

**Supplementary Content**

Supplemental Figure 1. Comparison of predicted values and real values of the ARIMA model.

Supplemental Figure 2. Comparison of predicted values and real values of the LSTM model

Supplemental Figure 3. Comparison of predicted values and real values of the ARIMA-LSTM model

Supplemental Figure 4. Comparison of predicted values and real values of the EEMD-LSTM model

This supplementary material has been provided by the authors to give readers additional information about their work.

Supplemental Figure 1. Comparison of predicted values and real values of the ARIMA model.

Supplemental Figure 2. Comparison of predicted values and real values of the LSTM model

Supplemental Figure 3. Comparison of predicted values and real values of the ARIMA-LSTM model

Supplemental Figure 4. Comparison of predicted values and real values of the EEMD-LSTM model
